# Supplementary material for: Refined CRISPR/Cas9 genome editing in the pea aphid uncovers the essential roles of Laccase2 in overwintering egg adaptation
Source: PLoS Genet. 2025 Jul 21;21(7):e1011557. doi: 10.1371/journal.pgen.1011557 (PMC12313077; doi:10.1371/journal.pgen.1011557)
Supplement: S1 Text — (PDF) [file pgen.1011557.s001.pdf]

S1 Text

IVT gRNA-mediated CRISPR/Cas9 genome editing

We designed two guide RNAs (Lac2-92 and Lac2-155) and injected *in vitro*-transcribed (IVT) single guide RNA (sgRNA) with recombinant Cas9 protein into the pea aphid eggs. Among 666 eggs injected, 368 (55.3%) turned to black, which is an indicator of healthy egg condition after microinjection. To estimate somatic mutation rates, we randomly picked up a small subset of the surviving eggs and subjected to deep sequencing. Genome DNA purified from each egg was barcoded in making Illumina amplicon-seq library and the somatic mutation rate was assessed individually. Among the 43 eggs surveyed, indels were detected (%indels > 1.0) in the genomes extracted from 22 eggs (51.1%), but the somatic mutation rate was low with a mean of 4.6% (Fig 4A).
