# Supplementary material for: Refined CRISPR/Cas9 genome editing in the pea aphid uncovers the essential roles of Laccase2 in overwintering egg adaptation
Source: PLoS Genet. 2025 Jul 21;21(7):e1011557. doi: 10.1371/journal.pgen.1011557 (PMC12313077; doi:10.1371/journal.pgen.1011557)
Supplement: S2 Text — (PDF) [file pgen.1011557.s002.pdf]

S2 Text

Optimization of DIPA-CRISPR in oviparous aphids

We injected Cas9 RNPs at different adult ages (day 0 to day eight post-emergence) to assess the optimal injection timing. Injected females were mated with males, and healthy black fertilized eggs were collected individually and analyzed via amplicon sequencing. No significant differences in indel rates were observed across the different adult stages, suggesting no specific period particularly susceptible to Cas9 RNP uptake within the first eight days after adult emergence (Fig 8C). However, the overall low indel rates indicate that further optimization may be necessary. Additionally, we evaluated the impact of the injection site on genome editing efficiency. Cas9 RNPs were injected into the thorax (base of the third thoracic legs) or the central abdomen (excluding the midline) of oviparous females. Eggs were collected at 2-day intervals for eight days post-injection and analyzed individually via amplicon sequencing. Injections into the abdomen resulted in significantly higher indel rates compared to thoracic injections for eggs collected on days 3-4. Although no significant differences were observed on days 5-6 and 7-8, higher indel rates were consistently observed with abdominal injections (Fig 8D).
