# Supplementary material for: Refined CRISPR/Cas9 genome editing in the pea aphid uncovers the essential roles of Laccase2 in overwintering egg adaptation
Source: PLoS Genet. 2025 Jul 21;21(7):e1011557. doi: 10.1371/journal.pgen.1011557 (PMC12313077; doi:10.1371/journal.pgen.1011557)
Supplement: S1 Fig — (PDF) [file pgen.1011557.s003.pdf]

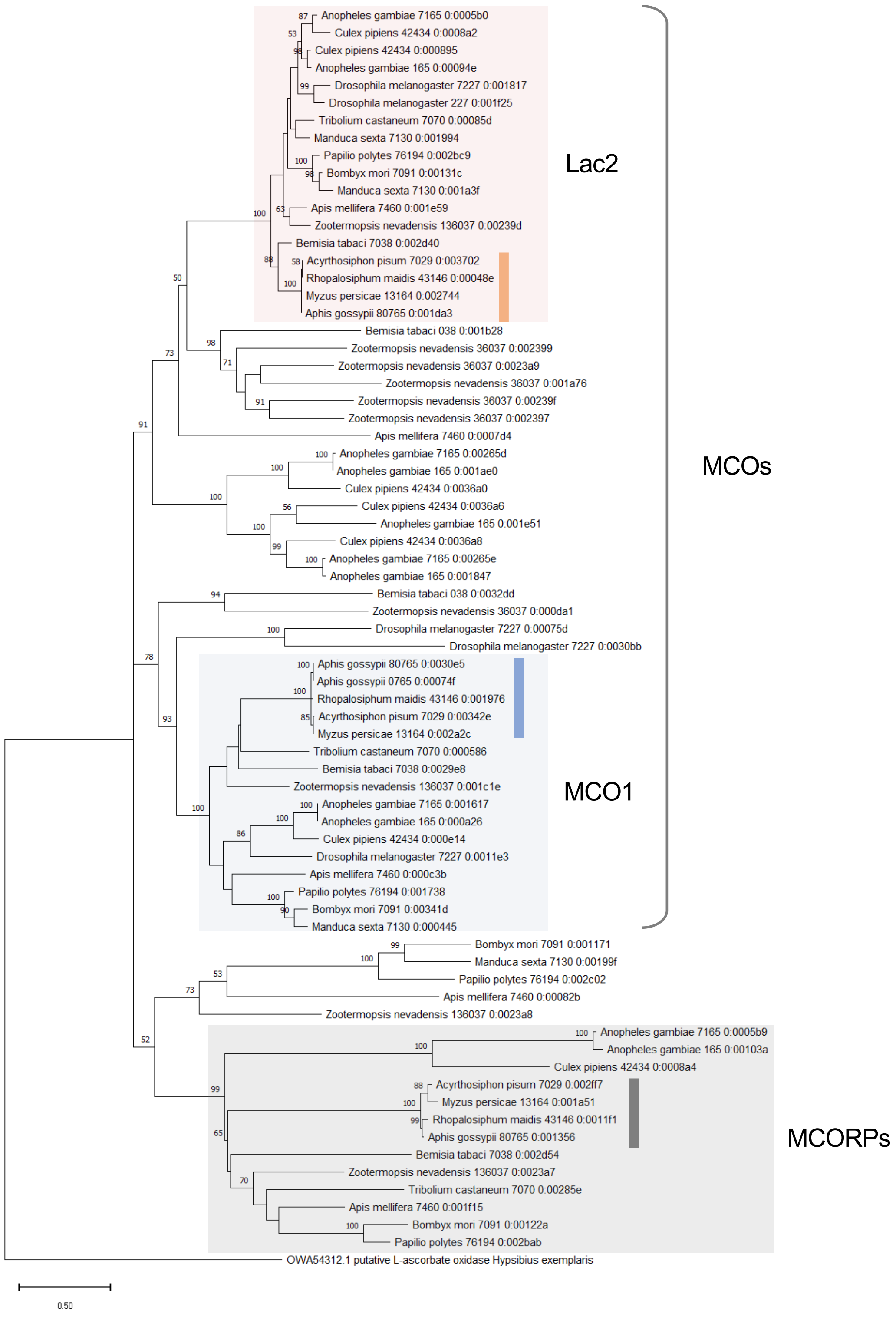

S1 Fig. Phylogenetic tree of multicopper oxidases (MCOs) and multicopper oxidase related proteins (MCORPs). The phylogenetic tree was constructed using the maximum likelihood (ML) method with the LG substitution model. Branch lengths are proportional to the extent of sequence divergence, and the tree is rooted with the L-ascorbate oxidase sequence from *Hypsibius exemplaris*, a species of tardigrade. Bootstrap values above 50% (based on 200 replicates) are shown at the corresponding nodes. The scale bar represents the number of substitutions per site. A total of 72 sequences were analyzed, including ingroup sequences retrieved from OrthoDB (https://www.orthodb.org/) at the Arthropoda taxonomic level, with their accession numbers provided in parentheses. The tree was generated using MEGA11 software. MCOs including MCO1 and Laccase 2 (Lac2), and MCORPs from aphid species are highlighted with orange, blue, and gray bars, respectively.
