# Supplementary material for: Refined CRISPR/Cas9 genome editing in the pea aphid uncovers the essential roles of Laccase2 in overwintering egg adaptation
Source: PLoS Genet. 2025 Jul 21;21(7):e1011557. doi: 10.1371/journal.pgen.1011557 (PMC12313077; doi:10.1371/journal.pgen.1011557)
Supplement: S2 Fig — (PDF) [file pgen.1011557.s004.pdf]

A

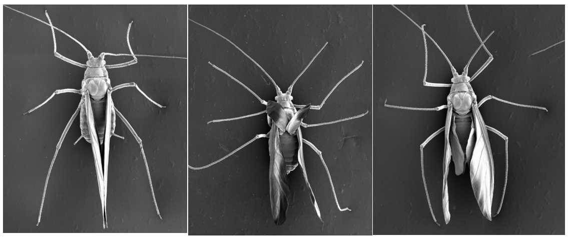

S2 Fig. Phenotype of heterozygous *Lac2* mutants. (A) SEM images of winged viviparous females induced from wild type and heterozygous *Lac2* mutant lines.
