# Supplementary material for: Refined CRISPR/Cas9 genome editing in the pea aphid uncovers the essential roles of Laccase2 in overwintering egg adaptation
Source: PLoS Genet. 2025 Jul 21;21(7):e1011557. doi: 10.1371/journal.pgen.1011557 (PMC12313077; doi:10.1371/journal.pgen.1011557)
Supplement: S3 Fig — (PDF) [file pgen.1011557.s005.pdf]

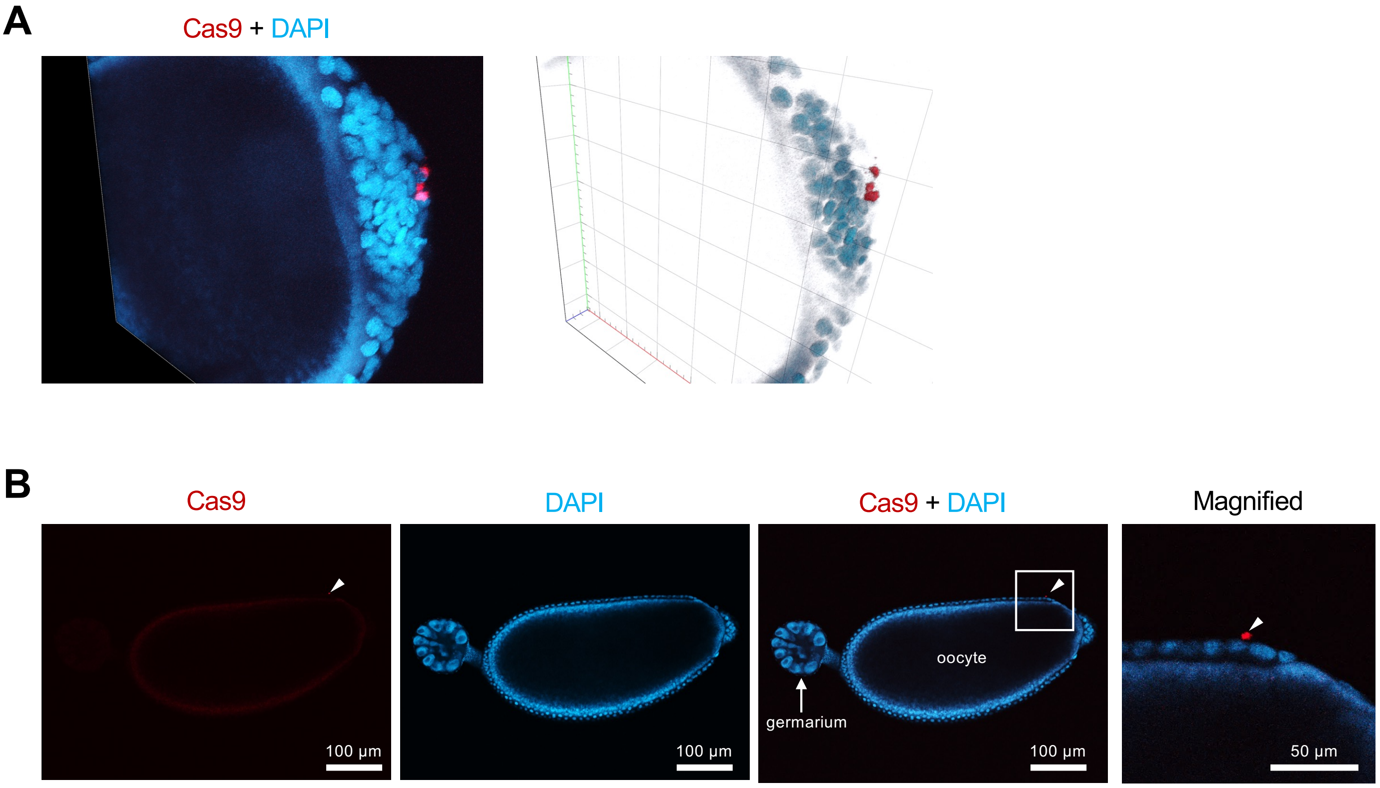

S3 Fig. Localization of Cas9 RNPs in oviparous aphid oocytes. (A) Z-stack imaging of Cas9 localization at the posterior pole of vitellogenic oocytes, showing multiple clustered signals. (B) Cas9 signals were detected on follicular cells along the lateral regions of vitellogenic oocytes.
