# Supplementary material for: Refined CRISPR/Cas9 genome editing in the pea aphid uncovers the essential roles of Laccase2 in overwintering egg adaptation
Source: PLoS Genet. 2025 Jul 21;21(7):e1011557. doi: 10.1371/journal.pgen.1011557 (PMC12313077; doi:10.1371/journal.pgen.1011557)
Supplement: Attachment — Submitted filename: R1_ResponseLett.v2.pdf [file pgen.1011557.s008.pdf]

Response to Reviewers

Manuscript Number: PGENETICS-D-24-01533

Refined CRISPR/Cas9 genome editing in the pea aphid uncovers the essential roles of *Laccase2* in overwintering egg adaptation

Shuji Shigenobu, Shinichi Yoda, Sonoko Ohsawa, Miyuzu Suzuki

We would like to express our profound gratitude for the professional advice and constructive comments given by the reviewers on this manuscript. In response, we carefully considered all the comments and revised the manuscript accordingly. In our view, these revisions have led to substantial improvements in the quality and clarity of the manuscript.

Before addressing each comment individually, we would like to provide an overview of the major revisions made to the manuscript, in addition to the many minor edits for overall improvement:

- The legend of Fig. 1 has been revised to provide more detailed information on the refined CRISPR/Cas9 protocol.
- Fig. 2A has been updated. While the previous version included only one sgRNA, the revised figure now shows all three sgRNAs used in this study.

The point-by-point response

Reviewer's Responses to Questions

Notes on the format in this response letter

- black: reviewers' comments
- blue: our answers
- *italic: texts quoted from manuscript*

Reviewer #1

[1-0]

Reviewer #1: This paper established an efficient genome editing system using CRISPR/Cas9 in the pea aphid targeting the Laccase2 gene. Since it has been challenging to edit the genome of aphids due to their complex life cycles, this system will provide useful information. It also provides important insights into the role of Laccase2 in aphids, especially on egg pigmentation, eggshell hardening and wing formation.

Overall, the experiments are well designed and carefully analyzed. On the other hand, the description is complicated due to the combination of several experiments, and some information is missing, making it difficult to follow. Also, the name of Laccase should be revised as it may cause confusion.

Ans) We sincerely thank the reviewer for the positive evaluation of our study and for recognizing its contributions to genome editing in aphids and the functional analysis of Laccase2. We also appreciate the constructive suggestions regarding the clarity of the manuscript. In the revised version, we have carefully addressed the reviewer's concerns and improved the organization and clarity of the descriptions. Specific revisions and clarifications are detailed below.

The following comments are provided mainly concerning the description of the manuscript.

[1-1]
1.
Laccase was originally distinguished into two groups, laccase1 and laccase2, but since laccase1 has no laccase activity, this name is no longer used and it is more appropriate to refer to it as MCO (see Dittmer & Kanost, 2010). The name Laccase2 can be left as it is, but please revise the description in the text as Laccase1 should be described as MCO1 in Fig. 2, Fig. S1, etc. Also, would the phylogenetic tree include MCORPs involved in ovarian development in Tribolium castaneum (Peng et al, 2014). Authors should also specify the relationship to MCORP when constructing the molecular phylogenetic tree.

Ans) We appreciate the reviewer's insightful comment and the helpful references provided. Following the clarification in Dittmer & Kanost (2010), we acknowledge that the term "Laccase1" is outdated, as this group lacks demonstrated laccase enzymatic activity. Accordingly, we have revised the terminology throughout the manuscript to refer to this group as "MCO1" rather than "Laccase1." This change has been implemented consistently, including in the figure legends and labels for Fig. 2 and Fig. S1.

We thank the reviewer for this valuable suggestion. During the revision process, we discovered and corrected a labeling error involving Lac1 (MCO1) and Lac2 in the original version of Supplementary Figure S1. In line with the reviewer's recommendation, we have also included representative MCORP sequences in the phylogenetic analysis. This revision allowed us to more clearly resolve the relationships among the MCO gene groups, specifically MCO1, Lac2, and MCORPs. As shown in the updated Supplementary Figure S1, the revised phylogenetic tree now clearly demonstrates that aphid species, including the pea aphid, possess three distinct MCO gene sets: MCO1, Lac2, and MCORP. We have revised the figure legend and Results section accordingly to clarify the placement and functional context of the MCORP group. Notably, unlike in Diptera and Blattodea, no lineage-specific expansions of MCO genes, beyond the conserved sets of MCO1, Lac2, and MCORP, were detected in aphids, suggesting a lack of order-level gene diversification in this lineage. We have revised the relevant descriptions in the Methods (Lines 487-493) and Results sections (Lines 568-582) to reflect this finding.

> *[L487] At the Arthropoda level, in addition to Laccase genes, multicopper oxidase-related proteins (MCORPs) were also retrieved. To identify MCORPs, we used the amino acid sequence previously reported as an MCORP in T. castaneum (NCBI accession: KJ500311; Peng et al., 2014) as a query in OrthoDB. This search identified the T. castaneum sequence 7070_0:002c8d,*

which is identical to 070_0:002c8d, as the corresponding MCORP. From the same 14 species listed above, 16 additional full-length MCORP amino acid sequences were retrieved.

[L584] Most insects, including the four aphid species analyzed, possessed two Laccase genes that clustered into two distinct clades corresponding to MCO1 (Lac1) and Lac2. Clustering showed strong support with high sequence conservation within these clades among aphids (bootstrap value = 100) (Fig 2C). Next, to expand the phylogenetic context of our analysis, we retrieved 71 MCO-related sequences at the broader Arthropoda level from OrthoDB, including not only Laccase genes but also MCORP sequences. Representative MCORPs were identified using the previously reported MCORP protein from Tribolium castaneum (NCBI accession: KJ500311; Peng et al., 2014) as a query. The L-ascorbate oxidase gene from the tardigrade Hypsibius exemplaris was used as an outgroup. The phylogenetic tree (S1 Fig) showed that the four aphid species consistently retain three distinct MCO gene groups: MCO1, Lac2, and MCORP. In contrast, other insect lineages such as Culex pipiens exhibit extensive lineage-specific gene expansions, with up to seven MCO genes spanning multiple clades. In aphids, however, MCO-related sequences were confined to their respective clades without evidence of recent gene duplications. This conserved MCO-related gene repertoire in aphids suggests a lack of order-level diversification and highlights the evolutionary stability and likely functional significance of these genes within the aphid lineage.

[1–2]
2. The author addresses the issue of Complex life cycle and Inbreeding depression on Page 27, but please describe what specific conditions were used. It is unclear how the authors set the day length and temperature for the summer and winter conditions. Also, the authors noted that they combined eight aphid lines, please specify the actual results and which combination was selected as the better one.

Ans) We appreciate the reviewer's suggestion. In response, we have added specific details regarding the incubator settings used to mimic summer and winter conditions. We have also provided additional information on the aphid strains and the specific combinations selected based on fertility and hatching rates. As these methodological details are described in our recent publication (Matsuda et al., 2024), we have cited this reference ([26]) in the revised manuscript.

[L667] Complex life cycle: To ensure a constant supply of eggs, we established a workflow using multiple incubators with varying conditions that mimicked summer (16 h light: 8h dark cycle) and winter (8 h light: 16 h dark cycle), allowing the transfer of aphids to induce sexual reproduction.

Inbreeding depression: Our preliminary tests revealed a severe inbreeding depression in pea aphids [26]. Therefore, we collected nine aphid lines from various host plants (alfalfa Medicago sativa, red clover Trifolium pratense, Vicia cracca, and Melilotus spp.) and identified pairs that exhibited high hatching rates and fertility. Based on these results, we selected two outbred combinations, ApL (female) × 0903A (male) and ApL (male) × KNb, for use in subsequent experiments [26].

[1-3]
3. Please specify if the sgRNA for IVT was designed in the same position as for Alt-R. If it was designed in a different position, this may also affect the results. Is the crRNA used for DIPA-CRISPR the same Lac2-4976BN used for the egg injection? Please specify how it was prepared.

Ans) We are sorry that the essential information was missing in the previous manuscript. Two sgRNAs generated by IVT, Lac2-92 and Lac2-155, were depicted in the updated version of Fig 2A. IVT Lac2-92 sgRNA and Alt-R 4976 were designed in the same position. Its figure legend was also updated accordingly as follows:

> *[L543] A close-up view of exon 4 highlights the target sites for CRISPR/Cas9-mediated gene knockout, with the designed sgRNAs and corresponding PAM sequences indicated. sgRNAs 4976BN and 4976AB were synthesized using the Alt-R CRISPR-Cas9 system (IDT), while Lac2-92 and Lac2-155 were prepared via in vitro transcription. Notably, 4976BN and Lac2-92 target the same genomic position. The position of the Cu-oxidase_3 domain (PF07732) within Lac2 is also marked.*

We also clarified this point in the legend for Fig 4:

> *[L699] See Fig 2A for the target position of each sgRNA. Notably, although 4976BN and Lac2-92 target the same genomic site, 4976BN induced a significant high indel frequency*

Regarding the DIPA-CRISPR experiments, we confirm that the same sgRNA (Lac2-4976BN) was used. To clarify this, we made the following textual revisions:

> *[L943] We injected Cas9 RNPs targeting Lac2 (sgRNA: Lac2-4976BN) into the body cavities of oviparous female aphids and examined indel mutation rates in the eggs they laid*
> *[L392] The injection solution, targeting the Lac2 locus (5'-GCTGTGAATCGTATGTTGCC-3') (Lac2-4976BN), was prepared by mixing 1.8 µL of 10 µM crRNA/tracrRNA duplex and*

Other minor comments are as follows.

[1-4]
>Title
pea aphids -> the pea aphid

Ans) We have changed as suggested. [L1]

[1-5]
>Page 8, line 175
Please describe the full name of AEL.

Ans) We have added the full description as suggested. [L189]

[1-6]
>Page 19, line 439
Is “The statistical” a typo?

Ans) They were unnecessary words. We removed them in this version. [L470]

[1-7]
>Page 29, line 662
What exactly is the “appropriate humidity level”?

Ans) To clarify this issue, we have added a more detailed description of the moist chamber setup in the Materials and Methods section. Specifically, we describe the use of a Ziploc container with a damp Kim Towel to create a high-humidity environment as follows. As described below, the relative humidity level is nearly 100% (saturated).

> *[L285] The moist chamber was prepared as follows: a Ziploc container was used as the chamber base. A Kim Towel was moistened with tap water, wrung out to remove excess water, and placed at the bottom of the container. The glass slides containing injected eggs were arranged in a slide holder and placed on top of the damp Kim Towel. The container was then sealed with its lid to maintain a high-humidity environment throughout incubation. Under these conditions, the relative humidity was maintained to be nearly 100%. The chamber was regularly monitored to ensure the high humidity and to prevent desiccation and fungal infection.*

>Page 32, line 758
Please add arrows to the legs in Fig. 6A.

Ans) As suggested, we have added arrows that indicate representative legs showing remarkable different pigmentation levels between the wild-type and the heterozygous mutant. We also updated the figure legend accordingly.

> *[L825] Grey arrows indicate representative legs showing remarkable different pigmentation levels between the wild-type and the heterozygous mutant.*

>Page 33, line 761
Fig 3A -> Fig 3B

Ans) Corrected as suggested. [L816]

>Page 33, line 780
appendages -> legs (?)

Ans) Changed as suggested. [L838]

>Fig 1
Please describe the conditions of the day length in detail.

Ans) We added the detailed information in the figure legend of Fig 1.

> *[L177] Sexual reproduction is induced by rearing aphids under a short-day photoperiod (8 h light / 16 h dark) at 15 °C, while the laboratory stock is maintained in a parthenogenetic, viviparous state under a long-day photoperiod (16 h light / 8 h dark) at 16 °C.*

>Fig 2A
The authors have designed two crRNAs, please specify which one.

Ans) We have updated Fig 2A so that both two crRNAs are shown in the figure.

>Fig 5C
Please add arrows to indicate red eyes.

Ans) As suggested, we have added an arrowhead to indicate red eyes with corresponding figure legend added as follows.

> *[L786] A developed red eye, visible through the eggshell, is indicated by an arrowhead.*

>Fig 8 legend
What does (Yoda) mean?

Ans) This was an unnecessary word and removed in the revised manuscript.

>S3 Fig
This is important information and should be moved to the main figure.

Ans) We appreciate this valuable suggestion. In response, we have moved the original S3 Figure to the main figures, and it is now presented as Figure 8C–D in the revised manuscript.
//

Reviewer #2

[2-1]
Reviewer #2: Shigenobu et al. reported genome editing techniques in the pea aphid, which is a model organism in evolution, ecology, development, and agriculture. They developed a

knockout technique using microinjection into fertilized eggs, enabling more stable, efficient, and rapid mutant generation compared to conventional methods. Additionally, for the first time in aphids, they introduced the simpler DIPA-CRISPR method, which is easier than microinjection into eggs. Methods for 1) preparation of eggs for microinjection, 2) introduction of the exogenous materials into eggs or bodies and 3) genome editing must be developed in a species- and strain-specific manner. The authors have made very careful observations, preparations, and evaluations. These advancements in genetic manipulation technologies are expected to significantly enhance the utility of this aphid as a model organism.
A previous study by Le Trionnaire et al. on genome editing in aphids has already been published. The authors cited this paper and discussed the similarities, differences, and advantages of their method in the Discussion section. They rigorously evaluated the genotypic and phenotypic changes resulting from introducing mutations in the target lac2 gene, providing strong evidence to support their claim that this method is highly effective.
The primary concern of this reviewer is that despite being submitted as a Method paper, the study seems to place greater emphasis on the functional analysis of the lac2 gene in overwintering eggs. While this is the first functional analysis of lac2 in overwintering eggs, the role of lac2 in the pigmentation of eggs has already been reported in other insects, such as mosquitoes. This raises questions about the originality of the finding of the lac2 function in the aphid. Therefore, this reviewer suggests that the authors focus more on the development of genome editing methods.

Ans) We sincerely appreciate the reviewer's positive evaluation of our work, particularly regarding the genome editing techniques we developed for the pea aphid. We also share the reviewer's view that our protocol has the potential to advance the use of the pea aphid as a model organism in studies of evolution, ecology, development, and agriculture.

The reviewer raised a concern that the manuscript may place greater emphasis on the functional analysis of *Lac2* in overwintering eggs, potentially overshadowing its contribution as a methodological study. We fully understand and acknowledge this point. In fact, the key objectives of our study are twofold: (1) to establish and refine CRISPR/Cas9-based genome editing methods optimized for the unique biology of the pea aphid, and (2) to provide biological insights into the function of Lac2, particularly in the context of overwintering adaptation. We have indeed been mindful of the balance between these two aspects throughout the preparation of the manuscript. In response to this comment and others raised during the review process, we have further strengthened the methodological components of the manuscript in this revision. Specifically, we have added more detailed descriptions of the genome editing procedures, including egg preparation, injection conditions, and incubation protocols. We believe these additions help to better highlight the methodological advances and ensure that both the technical and biological contributions of the study are clearly presented and appropriately weighted.

Additionally, this reviewer recommends that the authors highlight the limitations of previous aphid genome editing techniques in the Introduction and describe how this study addresses and improves upon these issues.

Ans) We thank the reviewer for this valuable suggestion. In response, we have revised the Introduction to highlight the limitations of previous aphid genome editing studies and to clarify the specific issues our study aims to address. The following passage has been added:

> *[L125] By addressing these issues, a couple of recent studies have successfully demonstrated the knockouts of stylin-01 and DDC in pea aphids [23,24]. However, there remain opportunities for further improvement. For instance, post-diapause hatching rates, survival rates, and germline transmission efficiency could be enhanced. In addition, the full workflow spans more than seven months, making it relatively time- and labor-intensive. Therefore, the efficiency of obtaining mutants and reducing laborious procedures warrants further optimization to increase the reliability and scalability of these approaches. Such refinements of CRISPR/Cas9 workflows could facilitate the broader application of genome editing in pea aphids and enhance their utility as model organisms.*

Minor comments/suggestions

[2-2]
Line 399: Why did the authors choose the BCR3 gene as a control for Cas9 RNPs targeting? Please explain the reason and the gene's function.

Ans) We thank the reviewer for this important comment. As a control for the amplicon sequencing experiment, we selected an sgRNA targeting the *BCR3* gene based on two main criteria: (1) the gene is unrelated to the multicopper oxidase family, to which *Lac2* belongs, ensuring no sequence similarity between the target sites; and (2) our laboratory has an established pipeline for NHEJ-based knockout of *BCR3* via egg microinjection.

BCR3 is specifically expressed in bacteriocytes, specialized cells involved in endosymbiosis with *Buchnera*, as previously reported (Shigenobu et al., 2013). Our lab has been investigating its function using the CRISPR/Cas9 technique described in this manuscript. The *BCR3*-targeting sgRNA used in this study has been well-characterized in our lab and exhibits a high cutting efficiency, comparable to that of the *Lac2*-targeting sgRNA. We have also successfully generated knockout lines using this sgRNA via egg microinjection. Based on these considerations, we selected *BCR3* as a suitable control. The same PCR primers were used for amplicon sequencing as those in the *Lac2* knockdown experiments, allowing us to quantify potential off-target cleavage and background noise at the *Lac2* locus under identical conditions.

We have revised the manuscript accordingly to clarify these issues in the Methods (Lines 423-434) and Results sections (Lines 948-955).

> *[L423] As a control, we injected Cas9 RNPs targeting the BCR3 locus (5'-GCAACCACTGTAACATACGG-3') using the same procedure as for the Lac2-targeted experiments. BCR3 is specifically expressed in bacteriocytes, specialized cells involved in endosymbiosis with Buchnera (Shigenobu et al., 2013). The BCR3-targeting sgRNA exhibits high*

cutting efficiency, comparable to that of the Lac2-targeting sgRNA, and successfully generated knockout lines via egg microinjection. Results from these control experiments were compared with those from the Lac2-targeted experiments to evaluate the specificity and effectiveness of DIPA-CRISPR in oviparous aphids. The same PCR primers were used for amplicon sequencing as those in the Lac2-targeted experiments to quantify potential off-target cleavage and background noise at the Lac2 locus under identical conditions. Amplicon-seq of the BCR3 locus was conducted using four biological replicates.

[L948] As a control, Cas9 RNPs targeting the BCR3 locus were injected into 14 oviparous females. Among the 64 fertilized eggs obtained, four were randomly selected for amplicon sequencing. BCR3 is an aphid-specific gene unrelated to Lac2, and the corresponding sgRNA has been well characterized. No apparent phenotypic changes were observed in the laid eggs, and amplicon sequencing showed an indel rate close to 0% (0.018%), a level consistent with background PCR or sequencing errors rather than off-target activity (Schirmer et al., 2015)

[2-3]
Line 439: “The statistical” may be omitted.

Ans) Removed unnecessary words.

[2-4]
Line 530: It would be better to mention why the presence of the signal peptide is important for the Lac2 function.

(Ans) We appreciate the reviewer’s suggestion. We have revised the text to clarify the importance of the signal peptide. The presence of a signal peptide suggests that Lac2 is secreted into the extracellular space, which is essential for its role in cuticle sclerotization and melanization, as phenol oxidase activity must occur in the cuticle region. We have revised the sentence accordingly in the manuscript (Lines 570-574).

[L570] SignalP analysis predicted a signal peptide at the N-terminus, indicating that Lac2 was likely to be secreted into the extracellular space, which is consistent with its expected role in the cuticle where phenol oxidase activity is required for sclerotization and melanization.

[2-5]
Lines 675-677: The authors performed genotyping of the survived eggs. Please explain which stage of embryos were used for genotyping.

(Ans) We used eggs aged 5–37 days after egg laying (AEL) for genotyping. This information has been added to the manuscript as follows:

[L729] We then randomly selected a subset of the surviving eggs (5-37 days AEL) and conducted amplicon-seq analysis individually for 64 eggs.

[2-6]
Lines 708-709: How was the amount and ratio of Cas9, crRNA, and tracrRNA determined?

(Ans) We used Cas9:gRNA complex at a 1:1 molar ratio. To clarify it, we modified the text in Materials and Methods as follows

> *[L233] The annealed tracrRNA/crRNA (final concentration: 30 nM) was mixed with the Cas9 nuclease (Alt-R S.p. Cas9 Nuclease V3, IDT; cat no. 1081058) at a final concentration of approximately 30 nM and incubated at 25°C for 10 min, resulting in the formation of Cas9:gRNA complex at a 1:1 molar ratio.*

We also added information on the injection volume used:

> *[L278] The volume of RNP injected into the aphid eggs was estimated to be 0.01 – 0.05 µL.*

[2-7]
Lines 710-717: Six stable mutant lines were obtained from 15 Fundatrix. Please explain how many Fundatrix produced the mutant lines.

Ans) We appreciate the reviewer's comment. Unfortunately, we could not determine precisely how many fundatrices contributed to the generation of the mutant lines with germline mutations. Due to space constraints and to facilitate efficient screening, multiple fundatrices were housed together in a single case, allowing them to give birth collectively. As a result, it was not possible to associate individual mothers with specific mutant offspring. However, based on our experimental records and the distribution of the resulting mutations, we estimate that the six stable mutant lines were derived from two or three fundatrices.

[2-8]
Lines 755-760: mut-4F male showed reduced pigmentation. mut-4F and mut-1D showed winkled wings. Please explain the phenotypes of the other mutants that were not described in the manuscript. If the non-frameshift mutation in the mu-2B did not change the phenotypes in males, the importance of the frameshift mutation could be emphasized.

Ans) We thank the reviewer for this insightful comment. We agree that phenotypic analysis of the non-frameshift mutant line (mut-2B) would provide additional insights into the functional importance of the coding sequence at this locus. Unfortunately, the mut-2B line was lost during maintenance in the laboratory, and we are currently unable to assess its phenotype in detail. Based on our experimental records, no obvious wing abnormalities were observed in males of the mut-2B line, suggesting that this mutation may not have caused a noticeable wing phenotype. However, due to the uncertainty surrounding this observation and the absence of the line, we opted not to include this information in the manuscript.

[2-9]
Lines 885-886: How many control animals injected with BCL2 crRNA were prepared? What was the result of the control experiment?

Ans) We thank the reviewer's comment. As a control, we injected Cas9 RNPs targeting the BCR3 locus into 14 oviparous aphids. No phenotypic changes were observed in the eggs laid by these individuals, indicating that the BCR3-targeting sgRNA itself did not induce any nonspecific developmental abnormalities under our experimental conditions. We have clarified this point in the Results section (Lines 948–955).

> *[L948] As a control, Cas9 RNPs targeting the BCR3 locus were injected into 14 oviparous females. Among the 64 fertilized eggs obtained, four were randomly selected for amplicon sequencing. BCR3 is an aphid-specific gene unrelated to Lac2, and the corresponding sgRNA has been well characterized in our laboratory. No phenotypic changes were observed in the laid eggs, and amplicon sequencing showed an indel rate close to 0% (0.018%), a level consistent with background PCR or sequencing errors rather than off-target activity (Schirmer et al., 2015).*

[2-10]
Lines 894-896: The indel rates of eggs in the DIPA-CRISPR experiments were shown here. Were these eggs collected from the same mother or several different mothers? Because the authors injected Cas9 RNPs into nine adult females, information on the difference in the efficiency of DIPA-CRISPR between individuals helps to understand the reproducibility of this method.

Ans) We thank the reviewer for this important comment. The eggs analyzed by amplicon sequencing were collected from several different mothers. All nine injected oviparous females were reared together in the same plastic case, and the eggs laid by these individuals were pooled without identifying which mother laid which egg. Therefore, the reported indel rate represents the combined outcome across multiple individuals. We agree that analyzing the variability between individual mothers would provide useful insight into the reproducibility of the method, and we plan to address this in future experiments.

[2-11]
Lines 932-933: The two mosaic Lac2 mutants were from the same mother or two different mothers?

Ans) We thank the reviewer for this question. Unfortunately, we could not determine whether the two mosaic Lac2 mutants originated from the same mother or from different mothers. As noted in our previous response [2–10], the Lac2 Cas9-RNP–injected females were also reared together in a shared container. Therefore, the laid eggs could not be traced back to individual mothers

[2-12]
Line 1118: It would be nice for readers to know why asexual eggs are not useful for genome editing in the aphid.

Ans) We thank the reviewer for this comment. In aphids, asexual reproduction occurs via viviparity—females give birth to clonal live offspring without producing eggs. Therefore, there are no "asexual eggs" that can be targeted for microinjection-based genome editing. While genetic manipulation of asexual individuals would be highly advantageous—since it avoids the need for diapause and concerns about inbreeding—currently available genome editing approaches are limited to the sexually reproducing stage, which produces fertilized, oviparous eggs. Future studies may explore the application of methods like DIPA-CRISPR to asexual viviparous embryos, which could make gene editing in clonal lineages more feasible. We have added a brief explanation in the Discussions section to clarify this point.

> *[L1234] In aphids, asexual reproduction takes place through viviparity, meaning genetically identical offspring develop inside the mother and are delivered live instead of being laid as eggs. Therefore, conventional genome editing approaches that target externally laid eggs cannot be applied directly to the asexual generation. Although genome editing of asexual individuals would offer significant advantages, such as eliminating the need for diapause and avoiding issues related to inbreeding, its feasibility remains a challenge. Future work may explore the possibility of adapting DIPA-CRISPR for use in viviparous asexual females, potentially expanding the applicability of gene editing in aphid research.*

[2-13]
Fig 4D: There is no description of the difference in color of each line.

Ans) We thank the reviewer for pointing out this oversight. We have added a description of the color coding in the figure legend for Fig 4D, as follows:

> *[L707] (D) Indel distribution profiles from amplicon-seq of the three Lac2 crispant eggs shown in (C). The percentage of Illumina reads containing indels is plotted relative to the target Cas9 cut site (position 0). Green, blue, and red lines correspond to the first, second, and third egg from the left in panel (C), respectively.*

[2-14]
Fig 8 legend: (Yoda) may not be necessary.

Ans) Removed unnecessary words.

//

Reviewer #3

[3-1]
Reviewer #3: This paper reports (1) improved protocols using CRISPR/Cas9 with aphids and (2) an experimental analysis of the role of the laccase 2 gene in aphid biology.

Aphids are a prime model for developmental plasticity, in which the same genotype can produce a variety of distinct female morphs based on environmental cues during early development. Aphids are also a central model for heritable symbiosis, with some hundreds of papers on the aphid/Buchnera system. Finally, they are major crop pests and are used in studies of host plant recognition and specialization and in studies of speciation mediated by host plant shifts. The pea aphid has come to be the primary species used for studies on this group and is studied by labs around the world. It has a chromosome-level genome RefSeq assembly.

Despite their importance, there are relatively few experimental genetics studies on aphids due to the unique challenges of their life cycles, as detailed in this manuscript. RNAi has had only limited success due to the high RNase activity in aphid tissues. Germline modification is challenging as the asexual phase does not provide eggs that can be injected, and the sexual phase involves induction of sexual morphs (~2 months), mating them, then a long egg diapause (~ 3 months).

The protocols described here for CRISPR/Cas9 will be enormously useful. The manuscript describes everything a researcher would need to know for knocking out target genes in pea aphid, from how to continuously produce the sexual forms and achieve good levels of egg hatching to the details of designing and generating the CRISPR constructs and injection techniques themselves. The presentation of both an egg injection protocol and the DIPA-CRISPR protocol for injecting sexual mothers to obtain mutant progeny.

The findings on the Lac2 functions are well-supported and interesting, even if not very surprising. This was a good choice for demonstrating the efficacy of these methods, as it enabled visualization of the various outcomes such as the mosaic embryos of the DIPA-CRISPR methods (shown in Fig. 9).

I have no major suggestions for changing the manuscript. I have listed below some minor issues.

Ans) We sincerely appreciate the reviewer’s thoughtful and encouraging comments. We are pleased that the reviewer found both the methodological advancements and the biological findings of our study to be valuable. We have carefully addressed all of the issues raised, as detailed below.

Minor comments:
[3-2]

Line 575: Please define “AEL”

Ans) The definition of AEL (after egg laying) has been added at the indicated position in the legend of Fig 3 [L621].

[3-3]
Line 176: Please define “PGC”

Ans) The definition of PGC (primordial germ cell) has been added at the specified location in the text. [L190]

[3-4]
Line 182: I think the word “to” should be “from”

Ans) We agree with the reviewer’s observation. The wording has been corrected from “to” to “from” as suggested. [L197]

[3-5]
Line 268: What are the “appropriate humidity levels”? This information would be helpful for someone trying to use your methods.

Ans) We thank the reviewer for this helpful comment. In response, we have added a more detailed description of the moist chamber setup in the Materials and Methods section. Specifically, we describe the use of a Ziploc container with a damp Kim Towel to create a high-humidity environment as follows:

> *[L285] The moist chamber was prepared as follows: a Ziploc container was used as the chamber base. A Kim Towel was moistened with tap water, wrung out to remove excess water, and placed at the bottom of the container. The glass slides containing injected eggs were arranged in a slide holder and placed on top of the damp Kim Towel. The container was then sealed with its lid to maintain a high-humidity environment throughout incubation. Under these conditions, the relative humidity was maintained to be nearly 100%. The chamber was regularly monitored to ensure the high humidity and to prevent desiccation and fungal infection.*

We also included a measurement of the humidity level within the chamber. Under these conditions, the relative humidity was maintained at nearly 100% (saturated).

[3-6]
Line 575: Please define “AEL” in this figure legend.

Ans) The definition of AEL (after egg laying) has been added at the indicated position in the legend of Fig 3 [L621].

[3-7]
Line 639-641: Please add a citation for this statement abou nuclease resistance and reduced immune response.

Ans) The statement regarding nuclease resistance and reduced immune response is based on information provided by the manufacturer (IDT) for the Alt-R CRISPR/Cas9 system. Although we could not find a corresponding academic publication, we have added a link to the product website in the revised manuscript for reference:
https://sg.idtdna.com/pages/products/crispr-genome-editing/alt-r-crispr-cas9-system [L691].

[3-8]
Line 687: “indicate” should be “indicates”

Ans) Corrected the grammatical error as suggested. [L741]

[3-9]
Line 710: “Fundatrix” should be “Fundatrices”

Ans) Corrected the grammatical error as suggested. [L764]

[3-10]
Line 828: Tunning (typo)

Ans) Corrected the typo to “Tanning” [L886]

[3-11]
Lines 890-891: Please mention how many control experiments were conducted with BCR3, to give context to the “close to 0%” value. The “close to” qualification is surprising, as I would expect 0%. I wonder why there were any at all.

Ans) We conducted four biological replicates for Amplicon-seq of BCR3. In our control experiments targeting the BCR3 locus, we observed indel rates close to 0% (0.018%) at the *Lac2* locus. The rare low-frequency variants detected are likely due to PCR or sequencing artifacts rather than off-target activity. This is consistent with previously reported error rates for the Illumina MiSeq platform, where substitution errors occur at an average rate of 0.6–1.1%, and indels are approximately 100-fold less frequent. Schirmer et al. (2015) demonstrated that even bases with high-quality scores can occasionally carry errors due to inherent limitations in sequencing chemistry and sequence-specific biases. We have added this explanation in the Methods (Lines 431-434) and Results sections (Lines 948-955).

[L431] The same PCR primers were used for amplicon sequencing as those in the Lac2-targeted experiments to quantify potential off-target cleavage and background noise at the Lac2 locus under identical conditions. This control experiment was conducted using four biological replicates

[L948] As a control, Cas9 RNPs targeting the BCR3 locus were injected into 14 oviparous females. Among the 64 fertilized eggs obtained, four were randomly selected for amplicon sequencing. BCR3 is an aphid-specific gene unrelated to Lac2, and the corresponding sgRNA has been well characterized. No apparent phenotypic changes were observed in the laid eggs, and amplicon sequencing showed an indel rate close to 0% (0.018%), a level consistent with background PCR or sequencing errors rather than off-target activity (Schirmer et al., 2015).

[3-12]
Lines 1000-1016: having just read the results, this seems to simply repeat them. Perhaps it can be telescoped so that you can proceed more quickly to the discussion points?

Ans) Thank you for this valuable suggestion. We agree that the original text in this section was somewhat redundant with the Results and could be more concise. In the revised version, we have condensed the description to approximately half its original length, providing a more streamlined summary of the key findings. We believe this change improves readability. The revised text is as follows:

[L1063-1069] Our CRISPR/Cas9-mediated disruption of Lac2 revealed its essential roles in multiple developmental stages and morphs of the pea aphid. Homozygous knockouts resulted in pigment-less, soft-shelled eggs that were embryonically lethal (Fig 5C-F), while G0 and heterozygous mutants exhibited phenotypes such as reduced cuticle pigmentation, malformed wings, and molting defects (Fig 4C; Fig 6; Fig 9AB). These findings demonstrated the multiple critical roles of Lac2 in different developmental stages and morphs of the pea aphid.

[3-13]
Line 1112 “an another” (remove “an”)

Ans) Corrected the grammatical error as suggested. [L1165]

[3-14]
Line 1122 CRISPR/as9 (typo)

Ans) Corrected the typo as suggested. [L1175]
